# Development of rapid advice guideline and standard and continuous updating guideline: experiences and practice

**DOI:** 10.1186/s40779-021-00298-4

**Published:** 2021-02-03

**Authors:** Ying-Hui Jin, Xiao-Mei Yao, Xian-Tao Zeng

**Affiliations:** 1 Center for Evidence-Based and Translational Medicine, Zhongnan Hospital of Wuhan University, Wuhan, 430071 China; 2 Department of Health Research, Evidence and Impact, McMaster University, Hamilton, Ontario L8S 4L8 Canada; 3 Department of Urology, Zhongnan Hospital of Wuhan University, 169 Donghu Road, Wuchang District, Wuhan, 430071 China

**Keywords:** COVID-19, SARS-CoV-2, Rapid advice guideline, Clinical practice guideline, Evidence-based medicine

## Abstract

We published rapid advice guidelines and updated guidelines for coronavirus disease 2019 (COVID-19) management on February 6, 2020, and September 4, 2020, respectively. These two guidelines vary widely in their developmental background, type of evidence, grade of recommendation and so on. We shared our experience for the development of these two guidelines to help clinical practitioners better understand and implement guidelines and to help guideline developers facilitate communication and discussion for guideline development during the pandemic.

Dear Editor,

Early in 2020, during the novel coronavirus infection outbreak, we published a rapid advice guideline for the diagnosis and treatment of coronavirus disease 2019 (COVID-19; Guideline 1, https://mmrjournal.biomedcentral.com/articles/10.1186/s40779-020-0233-6#citeas) [1]. Later, in September 2020, we registered and published an updated guideline named “Chemoprophylaxis, diagnosis, treatments, and discharge management of COVID-19: An evidence-based clinical practice guideline (updated version)” (Guideline 2, https://mmrjournal.biomedcentral.com/articles/10.1186/s40779-020-00270-8) [2, 3]. These two guidelines were both prepared in accordance with the methodology and general rules of World Health Organization (WHO) Guideline Development [4]. The WHO Rapid Advice Guidelines advice was used in the development of Guideline 1, and the guideline was issued within 1 week. We convened multidisciplinary guideline development groups composed of health professionals (experts in respiratory medicine, infectious disease, critical care medicine, cardiology, emergency medicine, pediatrics, oncology, gerontology, laboratory medicine, medical imaging, clinical immunology, and clinical pharmacy) and methodologists for developing both guidelines.

For any newly identified infection, an absolute lack of direct evidence is the greatest challenge for guideline developers. Thus, for Guideline 1, we were totally reliant on indirect evidence, such as that for severe acute respiratory syndrome (SARS), Middle East respiratory syndrome (MERS), and influenza. Although research has reported that severe acute respiratory syndrome coronavirus 2 (SARS-CoV-2), which causes COVID-19, is similar to SARS-CoV (approximately 80% similar), the evidence for the possible benefits of treatment in patients with SARS or MERS disease was considered indirect because the patient populations, viruses, and possibly even drug effects were different. Therefore, the rating of the quality of evidence for all important efficacy outcomes was downgraded by two levels based on the Grading of Recommendations Assessment, Development and Evaluation (GRADE) approach [4]. Indirect evidence that played an important role in guideline development during the early stage of the epidemic gradually faded when direct evidence on COVID-19 appeared.

The COVID-19 pandemic is a rapidly changing situation. An increasing number of research papers are being published both in China and internationally, providing research evidence for managing COVID-19, which enabled direct evidence from COVID-19 patients to be used in the development of Guideline 2. Guideline 2 finally included 75 original papers (including 12 randomized controlled trials) and 33 systematic reviews or meta-analyses.

During the Guideline 1 development process, 11,500 symptomatic persons were screened, 276 were identified as suspected victims of infection, and 170 were diagnosed (including 33 in critical condition) as having COVID-19 by the guideline working group’s clinical professionals. Frontline clinicians have accumulated valuable experience in the diagnosis, treatment and nursing of COVID-19 patients. There was no direct research evidence to inform recommendations at that time, and these experiences were assessed as “expert evidence” for our guideline development. Expert evidence was highly valued in Guideline 1 development. We used a structured form to collect this information so that it could be aggregated and presented to the guideline panel in the summary of findings. Expert evidence can be solicited by examining case reports, summaries, and reports of topics. During the consensus process, if the evidence was agreed upon by more than 70% of frontline clinicians, it was considered high-quality evidence. However, “expert evidence” was used slightly differently in the development of Guideline 2. Expert evidence would not change the direction and strength of recommendations based on direct evidence, but it could influence questions that have very limited evidence and may form an “ungraded consensus-based statement” when more than 70% of working group members in the guideline panel believed this conclusion to be valid.

In the development of Guidelines 1 and 2, we adhered to the GRADE approaches and rules to assess the quality of a body of evidence [5], to develop and report recommendations, and to make some adjustments. First, assessments of the quality of the evidence that were not for pooled estimates of effect were available as a narrative synthesis of the evidence in the development of Guideline 1. Second, as COVID-19 spreads worldwide, there is an increasing number of ongoing trials, resulting in new research papers being published, possibly every day. We downgraded the quality of evidence for imprecision based on the threshold that represents the basis for a management decision rather than considering whether an optimal information size was reached. Third, rigorous search techniques were implemented in the development of Guideline 2; therefore, we thought the possibility of unidentified studies leading to publication bias was rare. Fourth, for diagnostic questions, studies measuring the impact of testing on patient-important or population-important outcomes were not available; hence, the guideline panels included only studies with diagnostic test accuracy outcomes, which were considered a surrogate outcome for patient-important benefits and harm. Last, despite very low evidence or only having expert evidence suggesting benefit in a life-threatening situation, strong recommendations may be warranted. For example, no clinical trials have demonstrated the benefit of the application of mechanical ventilation or its indications for use. However, as a common clinical life support and rescue method, mechanical ventilation is often used in life-threatening situations.

All guidelines need to be kept up to date and consistent with the best available evidence [6]. This is particularly important but difficult to achieve in the context of a public health emergency, such as COVID-19, when new data are constantly emerging and experience is continually accruing [7]. Although we updated Guideline 2 recently, its recommendations will require continuous updating in the future to incorporate increasingly high-quality direct evidence.

## Conclusions

Guidelines of all types should always be evidence-based. It is a common misunderstanding that evidence-based guidelines can be developed only if well- designed controlled trials exist. Recommendations are derived from a systematic review of evidence, which is the current best evidence, and guidelines in a pandemic are no exception. The guidelines should be updated with continuously emerging evidence.

## Data Availability

Not applicable.

## Abbreviations

COVID-19: Coronavirus disease 2019
GRADE: Grading of Recommendations Assessment, Development and Evaluation
MERS: Middle East respiratory syndrome
SARS: Severe acute respiratory syndrome
SARS-CoV-2: Severe acute respiratory syndrome coronavirus 2
